# Autism Spectrum Disorder- and/or Intellectual Disability-Associated Semaphorin-5A Exploits the Mechanism by Which Dock5 Signalosome Molecules Control Cell Shape

**DOI:** 10.3390/cimb46040194

**Published:** 2024-04-02

**Authors:** Miyu Okabe, Takanari Sato, Mikito Takahashi, Asahi Honjo, Maho Okawa, Miki Ishida, Mutsuko Kukimoto-Niino, Mikako Shirouzu, Yuki Miyamoto, Junji Yamauchi

**Affiliations:** 1 Laboratory of Molecular Neurology, Tokyo University of Pharmacy and Life Sciences, Tokyo 192-0392, Japan; s189014@toyaku.ac.jp (M.O.); miyamoto-y@ncchd.go.jp (Y.M.); 2 Laboratory for Protein Functional and Structural Biology, Center for Biosystems Dynamics Research, RIKEN, Yokohama 230-0045, Japan; kukimoto@riken.jp (M.K.-N.); mikako.shirouzu@riken.jp (M.S.); 3 Laboratory of Molecular Pharmacology, National Research Institute for Child Health and Development, Tokyo 157-8535, Japan; 4 Diabetic Neuropathy Project, Tokyo Metropolitan Institute of Medical Science, Tokyo 156-8506, Japan

**Keywords:** Sema5A, ASD, Dock5, Elmo2, signalosome, morphogenesis

## Abstract

Autism spectrum disorder (ASD) is a neurodevelopmental disorder that includes autism, Asperger’s syndrome, and pervasive developmental disorder. Individuals with ASD may exhibit difficulties in social interactions, communication challenges, repetitive behaviors, and restricted interests. While genetic mutations in individuals with ASD can either activate or inactivate the activities of the gene product, impacting neuronal morphogenesis and causing symptoms, the underlying mechanism remains to be fully established. Herein, for the first time, we report that genetically conserved Rac1 guanine-nucleotide exchange factor (GEF) Dock5 signalosome molecules control process elongation in the N1E-115 cell line, a model line capable of achieving neuronal morphological changes. The increased elongation phenotypes observed in ASD and intellectual disability (ID)-associated Semaphorin-5A (Sema5A) Arg676-to-Cys [p.R676C] were also mediated by Dock5 signalosome molecules. Indeed, knockdown of Dock5 using clustered regularly interspaced short palindromic repeat (CRISPR)/CasRx-based guide(g)RNA specifically recovered the mutated Sema5A-induced increase in process elongation in cells. Knockdown of Elmo2, an adaptor molecule of Dock5, also exhibited similar recovery. Comparable results were obtained when transfecting the interaction region of Dock5 with Elmo2. The activation of c-Jun N-terminal kinase (JNK), one of the primary signal transduction molecules underlying process elongation, was ameliorated by either their knockdown or transfection. These results suggest that the Dock5 signalosome comprises abnormal signaling involved in the process elongation induced by ASD- and ID-associated Sema5A. These molecules could be added to the list of potential therapeutic target molecules for abnormal neuronal morphogenesis in ASD at the molecular and cellular levels.

## 1. Introduction

During the development of the central nervous system (CNS), both neuronal and glial cells undergo continuous morphological changes [1,2]. In neuronal cells, morphogenesis comprises multiple stages [3,4], including the outgrowth of primitive neurites, elongation of neurites, navigation of neuronal processes, and the subsequent formation of synapses to form neuronal networks [3,4,5,6]. These processes are generated together with interactions with glial cells [5,6]. Neuronal plasticity, a key property, relies on the formation of flexible networks [5,6]. Although it is generally believed that the outgrowth of primitive neurites and their elongation represent the initial stages in the establishment of neuronal networks [3,4,5,6], the molecular mechanisms that govern changes in neuronal cell shapes, controlling neurite outgrowth and elongation, are not completely understood. Conversely, in neurological and neurodevelopmental diseases, cell morphogenesis can be particularly affected, especially at the early stage [5,6,7,8]. Aberrant cell morphogenesis in neuronal cells appears to be related to the onset or specific phenotype of neurological and neurodevelopmental diseases [3,4,5,6,7,8].

Individuals with autism spectrum disorder (ASD) encounter challenges in interpreting others’ thoughts and expressing their own thoughts through non-verbal means such as words, facial expressions, gaze, and gestures [9,10,11,12]. They can also exhibit a distinct inclination towards restricted interests and repetitive behaviors [9,10,11,12]. The collective term for conditions such as autism, pervasive developmental disorder, and Asperger’s syndrome is ASD [9,10,11,12]. ASD can be characterized as a neuronal developmental disorder that affects the integral sensory input and output system [9,10,11,12]. ASD is believed to be caused by genetic and/or environmental factors [13,14,15,16]. The majority of ASD cases (50–90%) are suggested to result from familial or sporadic (de novo) mutations [13,14,15,16]. The activities of the mutated gene products can be either activated or inactivated, resulting in gain-of-function or loss-of-function [13,14,15,16]. In either case, the gene mutations of ASD have multiple effects on neuronal and possibly glial cell morphogenesis during neurogenesis and neuritegenesis, particularly at developmental stages [13,14,15,16].

Semaphorin family molecules, which are mainly composed of the Sema domain, the plexin, semaphorin, and integrin (PSI) domain, and the thrombospondin (TSP) domain, are conserved transmembrane proteins found in both invertebrates and vertebrates. They function as axon guidance cues that respond to neuronal process elongation and navigation [17,18,19,20]. Increasing evidence illustrates the role of semaphorin family molecules in morphogenetic processes, including immunological responses, angiogenesis, cardiac and skeletal muscle morphological changes, and renal morphogenesis [17,18]. Semaphorins have different effects on different development periods, depending on their expression in different tissue and cell types [17,18]. Plexin family proteins have the function of binding to semaphorin family molecules and transmitting them into cells, especially for short-distance signaling [19,20]. Signals can also be transmitted in the opposite direction [17,18,19,20]. Each class of plexin (classes A to D) has a different specificity for the respective semaphorins (semaphorins-3 to -7 in humans and mammals), meaning that it can specifically bind to one or more semaphorin protein(s) [19,20].

Semaphorin-5A (Sema5A) is thought to be one such axon guidance cue to bind to plexin-B3, acting as both a ligand and, occasionally, a receptor [21,22,23,24]. In the CNS, Sema5A is expressed in specific neuronal cells and, to a lesser extent, primitive cells of the oligodendrocyte lineage [22,23]. The gene is strongly associated with ASD [25,26,27,28] and intellectual disability (ID) primarily during the developmental period [25,26,27,28]. Certain mutations of the gene are associated with ASD and/or epilepsy [29,30]. Analyses using genetically modified mice have demonstrated that Sema5A is involved in connections between different neurons in certain brain regions [31,32]. Herein, we report that neurite-like process elongation induced by the ASD- and ID-associated Arg676-to-Cys (R676C) mutation [30] within the TSP domain of Sema5A is mediated by a signalosome, including Rac1 guanine-nucleotide exchange factor (GEF) Dock5 as the Rac1 activator, in the N1E-115 cell line [33,34,35,36]. This provides evidence for a unique group of molecules involved in potential therapeutic target pathways in Sema5A-related ASD at the molecular and cellular experimental levels.

## 2. Materials and Methods

### 2.1. Key Antibodies and Plasmids

The key antibodies used in this study are listed in Table 1, and the key plasmids are also detailed in the table.

### 2.2. Generation of Plasmids

Sense and antisense Dock5 and Elmo2 guide(g)RNAs (Fasmac, Atsugi, Japan) were annealed and inserted into the mammalian small RNA transcription vector pSINsi-mU6 (Takara Bio, Kyoto, Japan, Takara Bio Gene No. X06980) in accordance with the manufacturer’s instructions. The mammalian expression plasmid encoding CasRx, integrated into the clustered regularly interspaced short palindromic repeats (CRISPR)/CasRx system, was purchased from Addgene (Watertown, MA, USA; Addgene Gene No. 109049). The isolated N-terminal region (amino acids 1 to 71) of human Dock5 was chemically synthesized and fused with the mammalian expression vector pcDNA3.1-N-eGFP (GenScript, Piscataway, NJ, USA).

The R676C mutations of Sema5A were generated from the plasmids encoding mouse Sema5A (Addgene Gene No. 72161) using the PrimeStar Mutagenesis Basal kit (Takara Bio) in accordance with the manufacturer’s instructions [36].

### 2.3. RNA Isolation and Reverse Transcription–Polymerase Chain Reaction (RT-PCR)

Total RNA was isolated with the Isogen kit (Nippon Gene, Tokyo, Japan). RNA-derived cDNAs were generated using the PrimeScript RT Master Mix kit (Takara Bio) in accordance with the manufacturer’s instructions.

RT products were used for PCR amplification with Gflex DNA polymerase (Takara Bio) in accordance with the manufacturer’s instructions. Their PCR products were subjected into agarose gels.

### 2.4. Culture of the Cell Line, Generation of a Stable Clone, and Induction of Neurite-Like Process Elongation

The mouse N1E-115 cell line was kindly donated by Japan Health Science Foundation (Tokyo, Japan). The cells were cultured on cell culture dishes (Nunc brand of ThermoFisher Scientific, Waltham, MA, USA) in high-glucose Dulbecco’s modified Eagle medium (DMEM; Nacalai Tesque, Kyoto, Japan; Fujifilm, Tokyo, Japan), 10% heat-inactivated fetal bovine serum (FBS) (Gibco brand of ThermoFisher Scientific), and penicillin–streptomycin mixed solution (Nacalai Tesque) in accordance with the manufacturer’s instructions.

Stable clones harboring the wild-type (indicated as WT in the figures) Sema5A or the gene with the R676C mutation (indicated as R676C in figures) were selected in the presence of the antibiotic G418 (Nacalai Tesque) in accordance with the manufacturer’s instructions. Their cells were cultured without cryopreservation.

To induce neurite-like process elongation, the cells were cultured in DMEM and 1% FBS containing penicillin–streptomycin mixed solution in 5% CO_2_ at 37 °C for 2 days, unless otherwise indicated. Cells with processes exceeding two cell bodies in length were considered to be neurite-like process-bearing cells [36].

Under these conditions, the estimated percentage of attached cells incorporating trypan blue was less than 5% in each experiment.

### 2.5. Plasmid Transfection

The ScreenFect A transfection kit (Fujifilm) was used for plasmid transfection in accordance with the manufacturer’s instructions. The medium was replaced 4 h after transfection. Unless otherwise indicated, transfected cells were generally used for cell biological and biochemical experiments at 48 h or more after transfection.

Under these conditions, the estimated percentage of attached cells incorporating trypan blue was less than 7.5% in each experiment.

### 2.6. Polyacrylamide Gel Electrophoresis and Immunoblotting

Collected cells were lysed in lysis buffer [36,37,38]. Their centrifugally collected supernatants (20 mg per sample) were denatured in sample buffers (Fujifilm). They were separated on a sodium dodecylsulfate (SDS) polyacrylamide gel. The cell lysate-derived proteins or the resultant precipitates were separated using polyacrylamide gel and transferred to a polyvinylidene fluoride (PVDF) membrane (Fujifilm). They were blocked with Blocking One (Nacalai Tesque) and immunoblotted using the respective primary antibodies and peroxidase enzyme–conjugated secondary antibodies.

Enzymatically reactive bands were detected using CanoScan LIDE400 (Canon, Tokyo, Japan) and scanned using CanoScan software (ver. 2023, Canon). The blots shown in the figures are representative of 3 blots. We performed multiple sets of experiments and quantified other bands with one control’s band as 100% using Image J software (ver. Java 8, https://imagej.nih.gov/ accessed on 5 December 2023).

### 2.7. Affinity Precipitation Assay to Detect GTP-Bound, Active Rac1

Recombinant glutathione-s-transferase (GST)-tagged conserved Cdc42 and Rac interactive binding (CRIB) domain, which specifically binds to GTP-bound, active Rac1 as well as Cdc42, was generated by *E. coli*. GST-tagged CRIB (20 µg per sample) was mixed with glutathione-resin (ThermoFisher Scientific). The resin was washed with lysis buffer [36]. The supernatants (1 mg per sample) of the lysed cells were mixed with GST-CRIB-absorbed resin, collected via centrifugation, and washed with lysis buffer.

The washed resin was denatured in sample buffers and applied to polyacrylamide gel via immunoblotting to detect GTP-bound, active Rac1.

### 2.8. Statistical Analysis

Values are shown as the means ± standard deviation (SD) of separate experiments. For intergroup comparisons, the unpaired Student’s *t*-test in Excel (ver. 2021, Microsoft, Redmond, WA, USA) was used.

If multiple comparisons were required, a one-way analysis of variance (ANOVA) followed by Tukey’s multiple comparisons test in Graph Pad Prism (ver. 5, GraphPad Software, San Diego, CA, USA) was used. Differences were considered statistically significant when *p* < 0.05.

### 2.9. Ethics Statement

The protocol approved by the Tokyo University of Pharmacy and Life Sciences Gene and Animal Care Committee (Approval Nos. LS28-20 and LSR3-011) was utilized for genetically modified cells and related techniques.

## 3. Results

### 3.1. Dock5 and Partner Elmo2 Are Involved in the Regulation of Process Elongation during Normal Morphogenesis

The increased process elongation induced by the ASD-associated Sema5A mutant protein was recovered through co-expression of the isolated RhoG-binding domain [36]. Therefore, we sought to identify which Dock-A subfamily molecule of the typical Dock family and the Elmo family molecule as the tight partner [39,40,41,42,43,44,45], acting downstream of RhoG, are present in N1E-115 cells. RT-PCR analyses revealed the specific detection of a Dock5 transcript of Dock-A members and an Elmo2 transcript of Elmo members (Figure S1). Next, we examined whether the Dock5 and Elmo2 signaling unit is actually involved in the regulation of process elongation under normal conditions. Knockdown of Dock5 using the CRISPR/CasRx-based specific gRNA (panel A of Figure S2) greatly decreased process elongation. Its knockdown also resulted in decreased expression levels of neuronal differentiation marker proteins growth-/growth cone-associated protein 43 (GAP43) and beta3 tubulin whereas control actin proteins remained comparable in control and Dock5-knocked down cells (Figure S3). Similarly, the knockdown of Elmo2 using the CRISPR/CasRx-based specific gRNA (panel B of Figure S2) decreased process elongation and the expression levels of neuronal differentiation marker proteins (Figures S4 and S5), demonstrating that the Dock5 and Elmo2 signaling unit specifically participates in neuronal morphological changes.

### 3.2. Dock5 and Elmo2 Are Required for Mutated Sema5A-Induced Process Elongation

**Figure 1:**
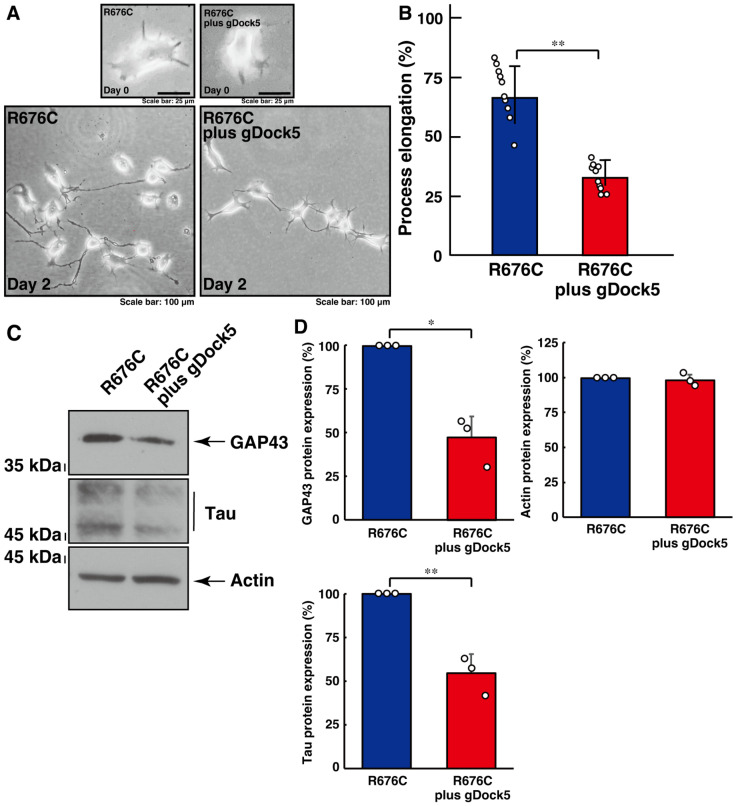
Knockdown of Dock5 using CRISPR/CasRx-based gRNA recovers mutated Sema5A-induced excessive process elongation. (**A**,**B**) N1E-115 cells harboring Sema5A with the ASD-associated R676C mutation were transfected with or without the plasmid encoding CasRx and gRNA specific for Dock5 and were allowed to differentiate for 0 or 2 days. Cells with processes exceeding a body length of two cells were counted as cells with neurite-like process elongation and are statistically depicted in the graph (** *p* < 0.01; *n* = 10 fields). (**C**,**D**) The lysates of cells at day 2 were immunoblotted with an antibody against a neuronal differentiation marker protein (GAP43 or Tau) or an internal control protein actin. Their immunoreactive band intensities are statistically depicted (** *p* < 0.01 and * *p* < 0.05; *n* = 3 blots).

We asked whether Dock5 contributes to the increased process elongation induced by Sema5A harboring the ASD-associated R676C mutation in N1E-115 cells (Figure S5). Knockdown of Dock5 using CRISPR/CasRx-based gRNA resulted in the recovery of increased process elongation by mutated Sema5A (Figure 1A,B). These results were accompanied by the recovered expression levels of neuronal differentiation marker proteins GAP43 and Tau (Figure 1C,D), indicating that mutated Sema5A-induced phenotypes are formed by Dock5.

**Figure 2:**
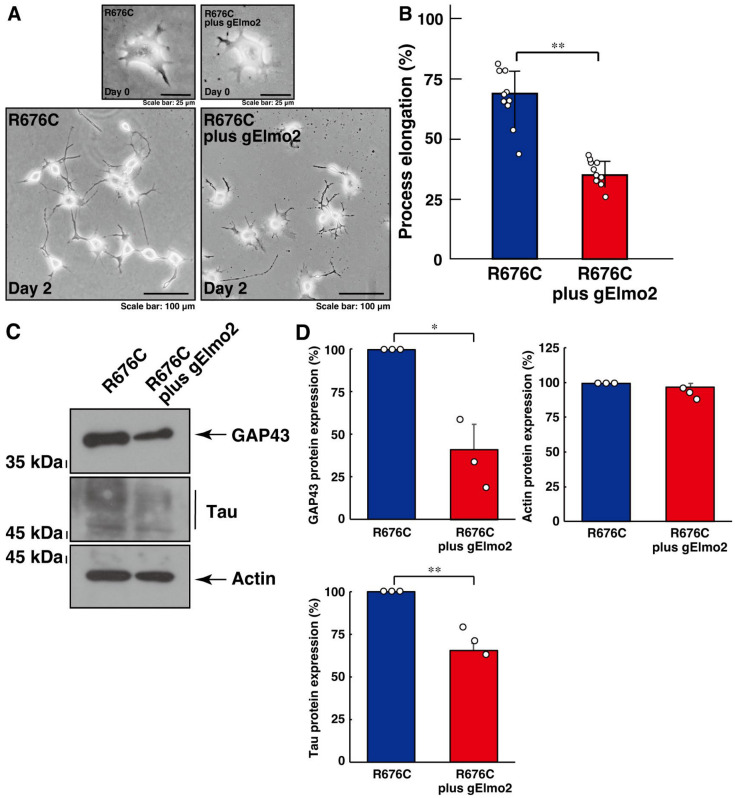
Knockdown of Elmo1 using CRISPR/CasRx-based gRNA recovers mutated Sema5A-induced excessive process elongation. (**A**,**B**) N1E-115 cells harboring Sema5A with the ASD-associated R676C mutation were transfected with or without the plasmid encoding CasRx and gRNA specific for Elmo1 and were allowed to differentiate for 0 or 2 days. Cells with processes exceeding a body length of two cells were counted as cells with neurite-like process elongation and are statistically depicted in the graph (** *p* < 0.01; *n* = 10 fields). (**C**,**D**) The lysates of cells at day 2 were immunoblotted with an antibody against a neuronal differentiation marker protein (GAP43 or Tau) or an internal control protein actin. Their immunoreactive band intensities are statistically depicted (** *p* < 0.01 and * *p* < 0.05; *n* = 3 blots).

Next, we examined the involvement of Elmo2 in the increased process elongation induced by Sema5A harboring the R676C mutation. Knockdown of Elmo2 led to the recovery of increased process elongation (Figure 2A,B) and increased expression levels of neuronal differentiation marker proteins (Figure 2C,D), indicating that the Dock5 and Elmo2 signaling unit mediates mutated Sema5A-induced phenotypes.

**Figure 3:**
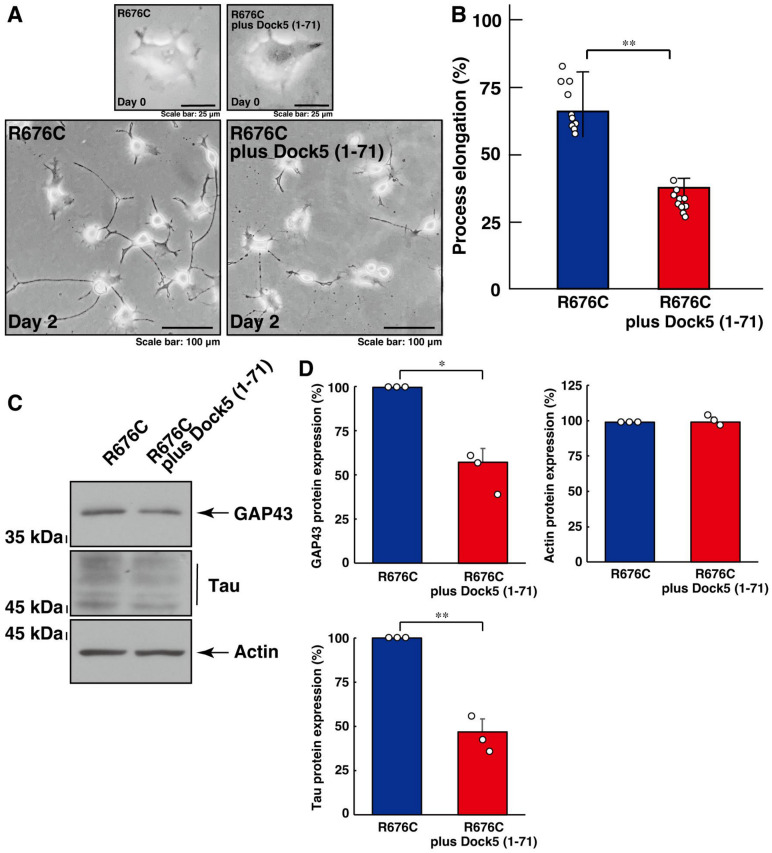
Transfection of an isolated binding region with Elmo members recovers mutated Sema5A-induced excessive process elongation. (**A**,**B**) N1E-115 cells harboring Sema5A with the ASD-associated R676C mutation were transfected with or without the plasmid encoding the Dock5 N-terminal region (amino acids 1 to 71) and were allowed to differentiate for 0 or 2 days. Cells with processes exceeding a body length of two cells were counted as cells with neurite-like process elongation and are statistically depicted in the graph (** *p* < 0.01; *n* = 10 fields). (**C**,**D**) The lysates of cells at day 2 were immunoblotted with an antibody against a neuronal differentiation marker protein (GAP43 or Tau) or an internal control protein actin. Their immunoreactive band intensities are statistically depicted (** *p* < 0.01 and * *p* < 0.05; *n* = 3 blots).

We further verified whether the interaction of Dock5 and Elmo2 is needed for the formation of increased process elongation phenotypes by Sema5A harboring the R676C mutation. We co-transfected the plasmid encoding the N-terminal region (amino acids 1 to 71) of Dock5, which provides a binding region with Elmo members [41,42,43,44,45], into cells. Dock5 (1-71) resulted in the recovery of increased process elongation (Figure 3A,B) and increased expression levels of neuronal differentiation marker proteins (Figure 3C,D), indicating that mutated Sema5A-induced phenotypes are formed by the interaction between Dock5 and Elmo2.

### 3.3. Rac1 Acts Downstream of Dock5 and Elmo2 in Mutated Sema5A-Induced Process Elongation

**Figure 4:**
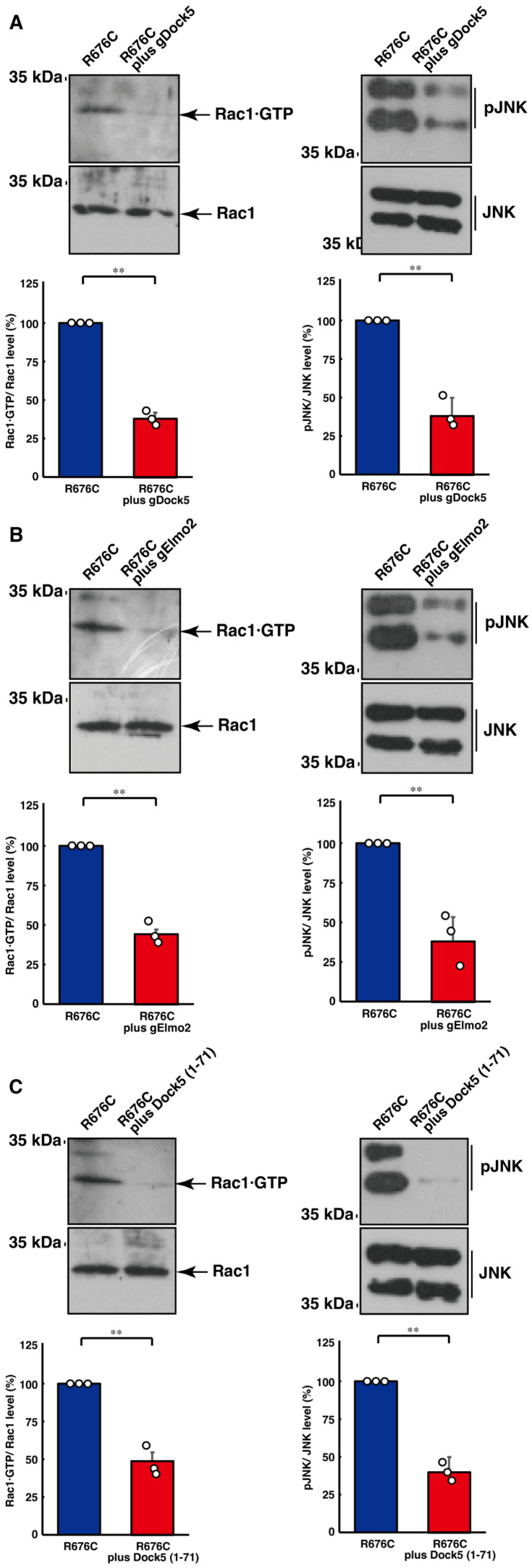
Transfection of Dock5 or Elmo2 gRNA or the Dock5 N-terminal region recovers Rac1 and JNK activated states induced by mutated Sema5A. (**A**,**B**) The transfected cell lysates in N1E-115 cells harboring Sema5A with the ASD-associated R676C mutation were used for the affinity precipitation assay to monitor GTP-bound Rac1 protein. The lysates were also subjected to immunoblotting with an antibody against Rac1. (**C**) The lysates were immunoblotted with an antibody against phosphorylated JNK or JNK. Their immunoreactive band intensities are statistically depicted (** *p* < 0.01; *n* = 3 blots).

Finally, we examined whether Rac1 actually acts downstream of Dock5 during the elongation of processes induced by Sema5A harboring the ASD-associated R676C mutation in N1E-115 cells. Knockdown of Dock5 decreased the levels of active GTP-bound Rac1, as determined by a pull-down assay using the isolated Rac1·GTP-binding domain. Similar results were obtained in the case of either knockdown of Elmo2 or co-expression of the N-terminal region of Dock5 (Figure 4A,B). Consequently, we explored whether the JNK cascade, a major Rac1 effector [41,42,43,44], actually acts downstream of Dock5 during the elongation of processes. Phosphorylated JNK, an active form, was decreased by knockdown of Dock5, Elmo2, or co-expression of the N-terminal region of Dock5 (Figure 4C). Taken together, these findings suggest that Rac1 and the effector cascade act downstream of Dock5 and Elmo2 in this potential pathological signaling.

## 4. Discussion

Genetic mutations in individuals with ASD can affect the cell morphology and function of neurons as well as the relationships between excitatory and inhibitory neurons, neurons and glial cells, and neurons and immune cells in the brain. The formation of ASD as a neurodevelopmental disorder is likely to involve the abnormal morphogenesis and developmental patterns of neurons [27,28]. The structure and function of Sema5A may be altered by amino acid substitutions in autism, affecting neuronal cell morphogenesis and morphologies [27,28]. Mutated Sema5A has been observed to exhibit increased elongation of processes in a neuronal cell line in an autocrine manner [36]. However, despite this relation of ASD-associated amino acid mutations with cell morphologies [36], it remains unclear what signaling pathways these mutations utilize to cause aberrant neuromorphological changes.

**Figure 5:**
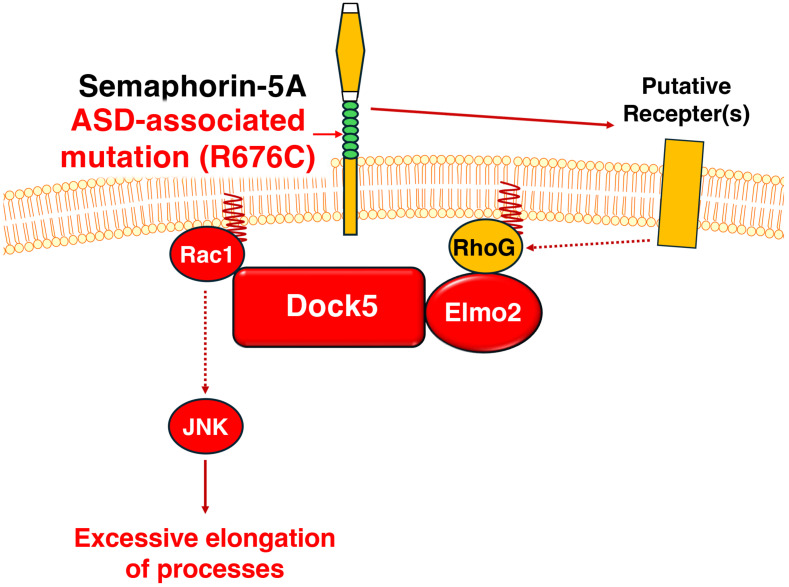
Schematic diagram of Dock5 signaling evoked by the ASD- and/or ID-associated Sema5A mutant protein underlying excessive elongation. Mutated Sema5A autocrinally regulates elongation of neurite-like processes through an unidentified putative receptor(s), which possibly transduces the signal to Elmo2 adaptor protein-mediated Dock5 GEF. The complex couples RhoG to Rac1 and the downstream JNK, enhancing excessive elongation.

Using the N1E-115 cell line as a model, we have identified that mutated Sema5A-induced neuromorphological changes, particularly process elongation, are mediated by signaling complexes containing Dock5. The promoting phenotypes are supported by the expression levels of neuronal differentiation markers, implying that the process elongation is related to changes in biochemical properties. In addition, the activities of Rac1, a downstream effector of Dock5, and the phosphorylation levels of JNK, recognized as neuronal differentiation markers, contribute to the mutated Sema5A-induced morphological changes. These results suggest that mutated Sema5A controls morphological changes by taking advantage of neuromorphogenesis-related intracellular signaling through Dock5 (see Figure 5).

In studies on human genetics, deletion of the short arm region of chromosome 5 is associated with phenotypic features such as microcephaly, ID, and Cri du chat syndrome in infancy [26]. Classical transcriptome analysis indicates that some transcripts, including the gene, are downregulated in some individuals with autism [25]. This deleted region includes the gene and some other genes, with studies associating it with causing infantile epilepsy [29,30]. Importantly, the respective mutations of Arg-676 and Ser-951 of Sema5A are critically associated with ASD and/or ID, probably depending on individual patients [27,28], suggesting that Sema5A is actually the responsible gene product of ASD and/or ID. These mutations occur between the extracellular and intracellular regions of Sema5a, allowing for potential disulfide bond formation in the extracellular region and a reducing group in the intracellular region within a predicted topological domain structure. The activities of ASD-associated mutant proteins generally involve activation or inactivation compared to the wild type [13,14,15,16]. In this case, Sema5A is activated by mutations at Arg-676 or Ser-951, and its mutation at Arg-676 is particularly active through effective cell surface transportation and/or secretion of Sema5A [36]. Sema5A acts autocrinally by interacting with an unknown receptor on the cell surface, suggesting a role as a ligand. In addition, since Sema5A is a bifunctional transmembrane protein, acting not only as a ligand but also as a receptor [21,22,23,24], this case may also involve effective cell surface transportation [36]. In either scenario, it is hypothesized that signaling through Dock5 underlies the effects of mutated Sema5A.

Dock family GEFs and Rac1 compose a genetically conserved molecular family of signaling pathways related to cell morphogenesis in developing stages, spanning from Nematoda to mammals but not to abnormal cell morphogenesis in disease states [39,40,41]. These molecules also trigger apoptosis during development [39,40,41]. In humans and rodents, Dock family molecules are divided into four subfamilies: Dock-A, -B. -C, and -D [42]. Dock5 is a member of the Rac1-GEF Dock-A subfamily within the typical Dock family [42]. Since Dock1 and Dock5 have multiple functions in various cell types, it is likely that the function of Dock5 is compensated by Dock1 [41,42,43,44,45,46].

Studies involving the mapping of naturally mutated mice with the rupture of lens cataract have identified the deletion of a continuous 27 nucleotides at the end of the 15th exon, which encodes the *dock5* gene in mouse chromosome 14. This deletion is identified as a candidate autosomal recessive disease gene [47]. The deletion results in decreased protein levels of Dock5, indicating that the loss-of-function of Dock5 is linked to abnormal morphogenesis in the formation of the eye lens, resulting in eye disease [47]. Thus, we asked whether and how Dock5 is involved in the regulation of morphogenesis in neuronal cells. The findings suggest that Dock5 is a specific signal transducer involved in both normal process elongation and mutated Sema5A-induced process elongation.

Elmo family molecules are well-established essential partners of all Dock-A family molecules [39,40,41,42,43,44,45]. Elmo1 exhibits wide expression profiles in the brain [48], and a similar expression pattern is observed for Elmo2. However, the expression levels of Elmo2 and Elmo1 differ depending on the respective brain and spinal cord regions [48]. It is unlikely that Elmo2 is completely compensated by Elmo1. In contrast to Dock5, it is well-known that Elmo2 positively regulates the elongation of neuronal dendrites in hippocampal neurons [49,50]. In addition, Elmo2 and Elmo1 play an essential role in the developmental stage when Sonic Hedgehog (Shh) guides commissural axons toward the floor plate [51]. It is suggested that Elmo2 responds to the Shh signal to control cytoskeletal remodeling, which underlies growth cone rotation [51]. Elmo2 and Elmo1 appear to act either specifically or cooperatively in controlling cytoskeletal proteins in neurons, depending on the respective extracellular signals; however, their partners are Dock3 and/or Dock4 [49,50,51]. Dock5 is unlikely to be involved in dendrite or commissural axon elongation. The formation of dendrites or axons by the signaling complex of Elmo2 or Elmo1 and Dock5 may depend on the type of neuron. It is possible that Elmo2 and Dock5 also exhibit specificity in certain types of neurological diseases.

On the other hand, JNK is a genetically conserved molecule of the signaling pathway related to cell morphogenesis during developmental stages from flies to mammals but not abnormal cell morphogenesis in disease states [52,53]. Like other members of the mitogen-activated protein kinase (MAPK) family, JNK is part of the signal complexes involved in the morphogenesis of almost all tissues and organs and is implicated in many diseases [52,53]. It is well-known that JNK kinase (JNKKK), an upstream kinase of JNK, is activated by Rac1 [52,53]. JNK itself phosphorylates cytoskeletal molecules and controls their activities [52,53]. The signaling coupling of Sema5A to Elmo2/Dock5 in both health and disease conditions may have specificity for process elongation in certain types of neuronal cells.

Plexin-B3 has been biochemically identified as the binding partner of Sema5A in neurons [54]. Plexin-B3, acting together with the interaction involving the Met proto-oncogene product (c-Met)’s receptor tyrosine kinase, links non-receptor tyrosine kinases c-Src and focal adhesion kinase (FAK) to small GTPases. This linkage controls cytoskeletal proteins, leading to cell morphological changes in cell types other than neuronal cells [55]. Given that Plexin-B3 is a multifunctional protein [17,18,19,20], Met could be one of the major signal transducers for Plexin-B3. Despite an unknown association of Plexin-B3 with ASD, it is of note that Met is recognized as a risk gene product for ASD [56,57]. It is also possible that signaling through Dock5 acts downstream of Plexin-B3 and c-Met.

The relationship between semaphorin family molecules and Alzheimer’s disease is increasingly recognized [58,59]. It is known that Sema3A is a component of extracellular matrix structures surrounding certain types of neuronal cells in the adult CNS. It plays a positive role in ending a critical period of plasticity [59]. Conversely, the failure of the system is believed to be one of the important factors contributing to Alzheimer’s disease-like phenotypes. Additionally, Sema5A and/or Sema7A are involved in the regulation of neurogenesis in adults, and dysfunction is also associated with Alzheimer’s disease-like phenotypes [58,59]. Genome-wide association studies (GWASs) suggest an association between single nucleotide polymorphisms (SNPs) of the gene and Alzheimer’s disease [60,61]. Further studies may reveal the association of Sema5A with many neurological diseases in addition to ASD and ID. Herein, we demonstrate that the increased process elongation induced by the Sema5A protein harboring the ASD-associated R676C mutation is mediated by signaling through Dock5. Knockdown of the respective molecules using CRISPR/CasRx-based gRNAs results in the recovery of increased process elongation. Additional studies may promote our understanding of not only the detailed mechanism by which mutated Sema5A intracellularly leads to the phosphorylation and activation of JNK via signaling through Dock5 but also the extent to which mutated Sema5A utilizes signaling through Dock5 and other signal transduction pathway molecules in increased process elongation, in both primary neuronal cells and genetically modified mice. These studies can allow us to clarify the relationship between the possible cellular phenotypes observed in this study and those of cells in patients. This line of investigation may facilitate a direct link between experimental results and potential therapeutic methods for Sema5A-associated ASD and ID and axon guidance molecule-related ASD.

## Figures and Tables

**Table 1:** Key materials used in this study.

Reagents or Materials	Companies or Sources	Cat. Nos.	Lot. Nos.	Concentrations Used
Key antibodies				
Anti-growth-associated protein 43 (GAP43)	Santa Cruz Biotechnology (Santa Cruz, CA, USA)	sc-17790	J0920	Immunoblotting (IB), 1:5000
Anti-beta III tubulin	Santa Cruz	sc-80005	H2721	IB, 1:5000
Anti-Tau	Santa Cruz Biotechnology	sc-21796	007	IB, 1:500
Anti-actin (also called pan-beta type actin)	MBL (Tokyo, Japan)	M177-3	J2521	IB, 1:5000
Anti-c-Jun N-terminal kinase (JNK, pan-JNK)	Santa Cruz Biotechnology	sc-7345	C1722	IB, 1:250
Anti-phospho-c-Jun N-terminal kinase (pJNK, pan-pJNK)	Santa Cruz Biotechnology	sc-6254	sc-6254	IB, 1:500
anti-Rac1	Proteintech (Rosemont, IL, USA)	66122-1-Ig	10011346	IB, 1:500
Anti-IgG (H + L chain) (rabbit) pAb-HRP	MBL	458	353	IF, 1:5000
Anti-IgG (H + L chain) (mouse) pAb-HRP	MBL	330	365	IF, 1:5000
**Recombinant DNAs**				
pCMV-SV40ori-mouse Sema5A-His	Addgene (Watertown, MA, USA)	72035	n.d.	1.25 microgram of DNA per 6 cm dish
pCMV-SV40ori-mouse Sema5A (R676C)-His	A construct generated using Cat. No. 72035 (Addgene)	derived from 72035	n.d.	1.25 microgram of DNA per 6 cm dish
CasRx (NLS-RfxCas13d-NLS)	Addgene	109049	n.d.	1.25 microgram of DNA per 6 cm dish
pSINsi-mU6-gRNA for mouse Dock5 (nucleotide numbers 1266 and 1410)	Generated in this study	n.d.	n.d.	1.25 microgram of DNA per 6 cm dish
pSINsi-mU6-gRNA for mouse Elmo2 (nucleotide numbers 909 and 1239)	Generated in this study	n.d.	n.d.	1.25 microgram of DNA per 6 cm dish
pcDNA3.1-N-eGFP-human Dock5 (amino acids 1 to 71 of the Elmo family molecule-binding region)	GenScript (Piscataway, NJ, USA)	n.d.	n.d.	1.25 microgram of DNA per 6 cm dish
pET42a-conserved Cdc42 and Rac interactive binding (CRIB) domain of human Pak1	see Pathophysiology 2023 30:548-566	n.d.	n.d.	5 micrograms of DNA per 1 litter size bacteria culture bottle

## Data Availability

The datasets used and/or analyzed in the current study are available from the corresponding author upon reasonable request.

## Supplementary Materials

The following supporting information can be downloaded at: https://www.mdpi.com/article/10.3390/cimb46040194/s1, Figure S1. Transcripts of typical Dock family members and their binding partner proteins. Figure S2. Effects of Dock5 or Elmo2 CRISPR/CasRx-based gRNA on knockdown in N1E-115 cells. Figure S3. Effects of Dock5 knockdown on process elongation in N1E-115 cells. Figure S4. Effects of Elmo2 knockdown on process elongation in N1E-115 cells. Figure S5. Effects of mutated Sema5A in process elongation in N1E-115 cells.
