# Supplementary material for: Autism Spectrum Disorder- and/or Intellectual Disability-Associated Semaphorin-5A Exploits the Mechanism by Which Dock5 Signalosome Molecules Control Cell Shape
Source: Curr Issues Mol Biol. 2024 Apr 2;46(4):3092–107. doi: 10.3390/cimb46040194 (PMC11049140; doi:10.3390/cimb46040194)
Supplement: Supplementary file 1 [file cimb-46-00194-s001.zip › cimb-2892538-supplementary.pdf]

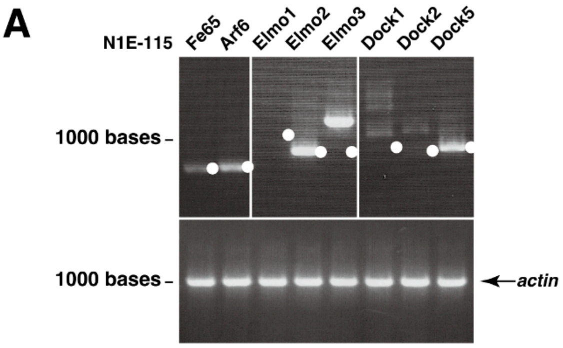

primers
sense-Fe65<br>TGGGTAGAGATGACCGAGGAG<br><br>antisense-Fe65<br>CTTGGAACTTCTGCACCAGCTC
sense-Arf6<br>ATGGGGAAGGTGCTATCCAAG<br><br>antisense-Arf6<br>TTAGGATTTGTAGTTAGAGGTTAACCATG
sense-Elmo1<br>ATGCAGAGTCAGAACCTAATAACAG<br><br>antisense-Elmo1<br>TCAGTTACAGTCATAGACAAAGTC
sense-Elmo2<br>ATGCCTTTGCACAGAAGCAC<br><br>antisense-Elmo2<br>CTGGAAATCATCCTGGCTCATC
sense-Elmo3<br>ATGAACCACCAGCTGCAAAC<br><br>antisense-Elmo3<br>CATAGGTGAGGGCATTCACTTTG
sense-Dock1<br>CAGGGGAGAATGACTTCCTTC<br><br>antisense-Dock1<br>CACCGTCCACCTTCATCAAC
sense-Dock2<br>ATGCCAAGAAGTGCACCCAG<br><br>antisense-Dock2<br>GGTGTCGATAAGAAGGAAGTGTCAG
sense-Dock5<br>TCAGCAAATTGCCATGGAGAC<br><br>antisense-Dock5<br>TGTCTTTGCTGGGTGTGAAG

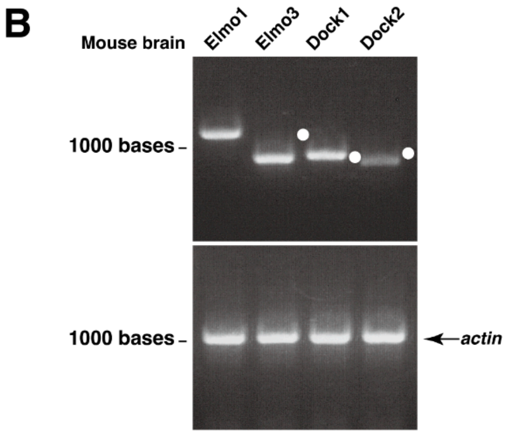

primers
sense-Elmo1<br>ATGCAGAGTCAGAACCTAATAACAG<br><br>antisense-Elmo1<br>TCAGTTACAGTCATAGACAAAGTC
sense-Elmo3<br>ATGAACCACCAGCTGCAAAC<br><br>antisense-Elmo3<br>CATAGGTGAGGGCATTCACTTTG
sense-Dock1<br>CAGGGGAGAATGACTTCCTTC<br><br>antisense-Dock1<br>CACCGTCCACCTTCATCAAC
sense-Dock2<br>ATGCCAAGAAGTGCACCCAG<br><br>antisense-Dock2<br>GGTGTCGATAAGAAGGAAGTGTCAG

Figure S1. Transcripts of typical Dock family members and their binding partner proteins in N1E-115 cells. (A, B) Total RNAs from cells were subjected to RT-PCR for Fe65, Arf6, Elmo family members Elmo1, Elmo2, and Elmo3, and Dock family members (Dock-A members) Dock1, Dock2, and Dock5. Dock family member–binding partners Fe65 and Arf6 are widely presenting transcripts that served as positive controls. Predicted lengths of the respective products are indicated by white circles. Actin transcripts were used as the internal control. Since Elmo1, Elmo3, Dock1, and Dock2 failed to be recognized as transcripts in N1E-115 cells, total RNAs from mouse brain tissues were used for RT-PCR. The primers' nucleotide sequences used in the respective experiments are also provided.

A

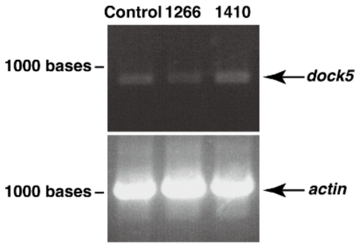

gRNA transcription sequences inserted into the plasmid
sense-BamHI-gDock5-1266-MluI-ClaI<br>gatccGCACCCGTGCAAAAATGCAGGGGTCTAAAACCCGTGCTATTGCTGTTGATCTGTTTTTat<br>antisense-BamHI-gDock5-1266-MluI-ClaI<br>cgatAAAAAACAGATCAACAGCAATAGCACGGGTTTTAGACCCCTGCATTTTTGCACGGGTGCg
sense-BamHI-gDock5-1410-MluI-ClaI<br>gatccGCACCCGTGCAAAAATGCAGGGGTCTAAAACGCCTTCCTCATCAAACACAGACTTTTTat<br>antisense-BamHI-gDock5-1410-MluI-ClaI<br>cgatAAAAAAGTCTGTGTTTGATGAGGAAGGCGTTTTAGACCCCTGCATTTTTGCACGGGTGCg

B

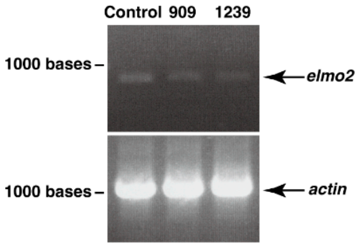

gRNA transcription sequences inserted into the plasmid
sense-BamHI-gElmo2-909-MluI-ClaI<br>gatccGCACCCGTGCAAAAATGCAGGGGTCTAAAACCTGGTCATTGGGATCCATCTTGTTTTTat<br>antisense-BamHI-gElmo2-909-MluI-ClaI<br>cgatAAAAAACAAGATGGATCCCAATGACCAGGTTTTAGACCCCTGCATTTTTGCACGGGTGCg
sense-BamHI-gElmo2-1239-MluI-ClaI<br>gatccGCACCCGTGCAAAAATGCAGGGGTCTAAAACGATCTCACAGAGCATCTTGGTGTTTTTat<br>antisense-BamHI-gElmo2-1239-MluI-ClaI<br>cgatAAAAAACACCAAGATGCTCTGTGAGATCGTTTTAGACCCCTGCATTTTTGCACGGGTGCg

Figure S2. Effects of Dock5 or Elmo2 CRISPR/CasRx-based gRNA on knockdown in N1E-115 cells. (A, B) The respective CRISPR/CasRx-based gRNAs (1266th or 1410th from A^1UG) for Dock5 were transfected with cells and subjected to RT-PCR with primers used to identify the respective transcripts. Since 1266th gRNA of Dock5 was more effective than 1410th gRNA, we selected 1266th gRNA for subsequent experiments. Actin transcripts were used as the internal control. (B) The respective CRISPR/CasRx-

based gRNAs (909th or 1239th from A[1]UG) for Elmo2 were transfected with cells and subjected to RT-PCR with primers used to identify the respective transcripts. Since 909th gRNA of Elmo2 was more effective than 1410th gRNA, we selected 909th gRNA for subsequent experiments. Actin transcripts were used as the internal control.

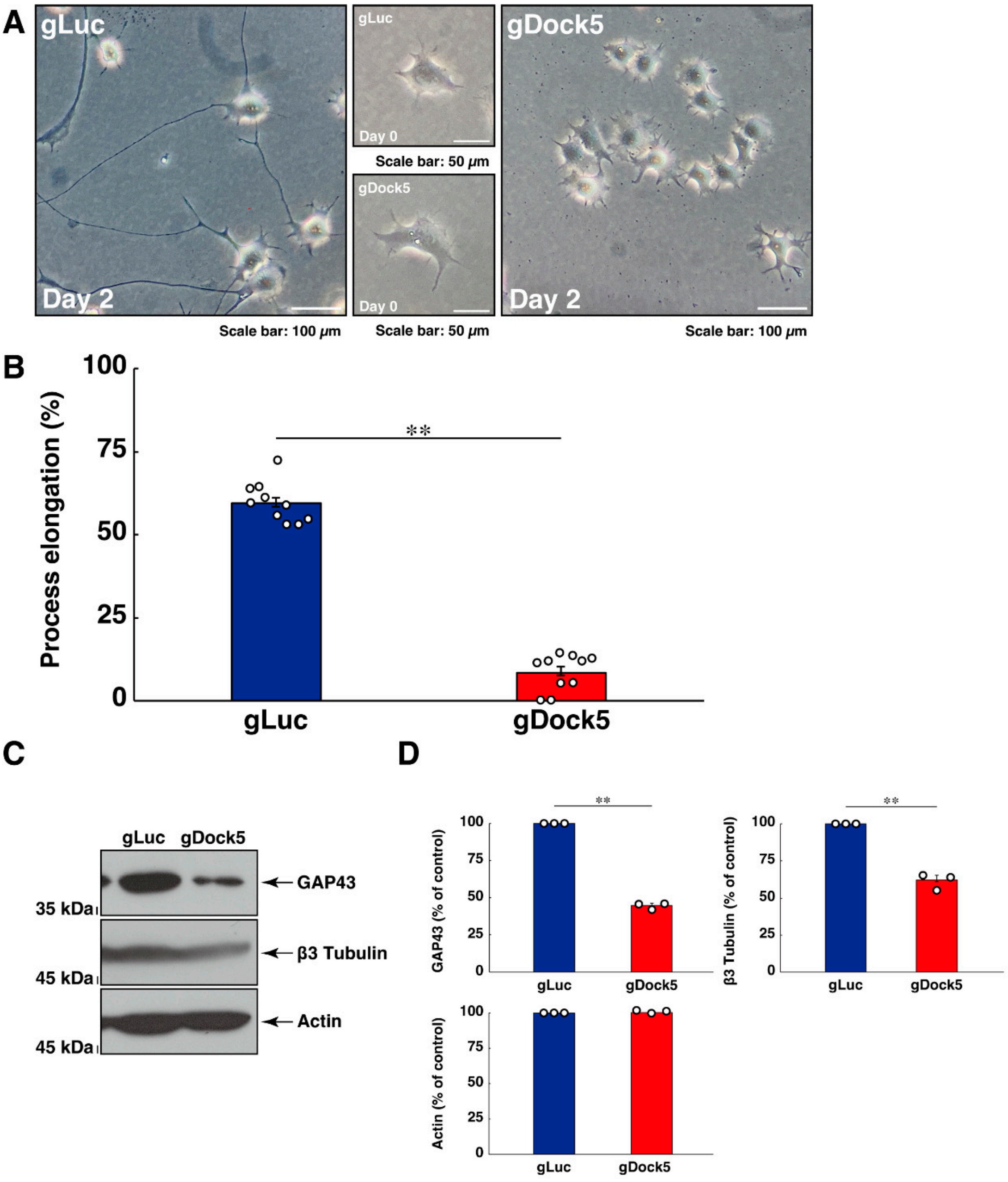

Figure S3. Effects of Dock5 knockdown on process elongation in N1E-115 cells. (A, B) (A, B) Cells were transfected with the plasmid encoding CasRx and gRNA specific for Dock5 or mock plasmid and allowed to differentiate for 0 or 2 days. Cells with processes exceeding a body length of two cells were counted as cells with neurite-like process elongation and statistically depicted in the graph (** $p < 0.01$; n = 10 fields). (C, D) The lysates of cells at day 2 were immunoblotted with an antibody against a neuronal differentiation marker protein (GAP43 or beta3 tubulin) or an internal control protein actin. Their immunoreactive band intensities are statistically depicted (** $p < 0.01$; n = 3 blots).

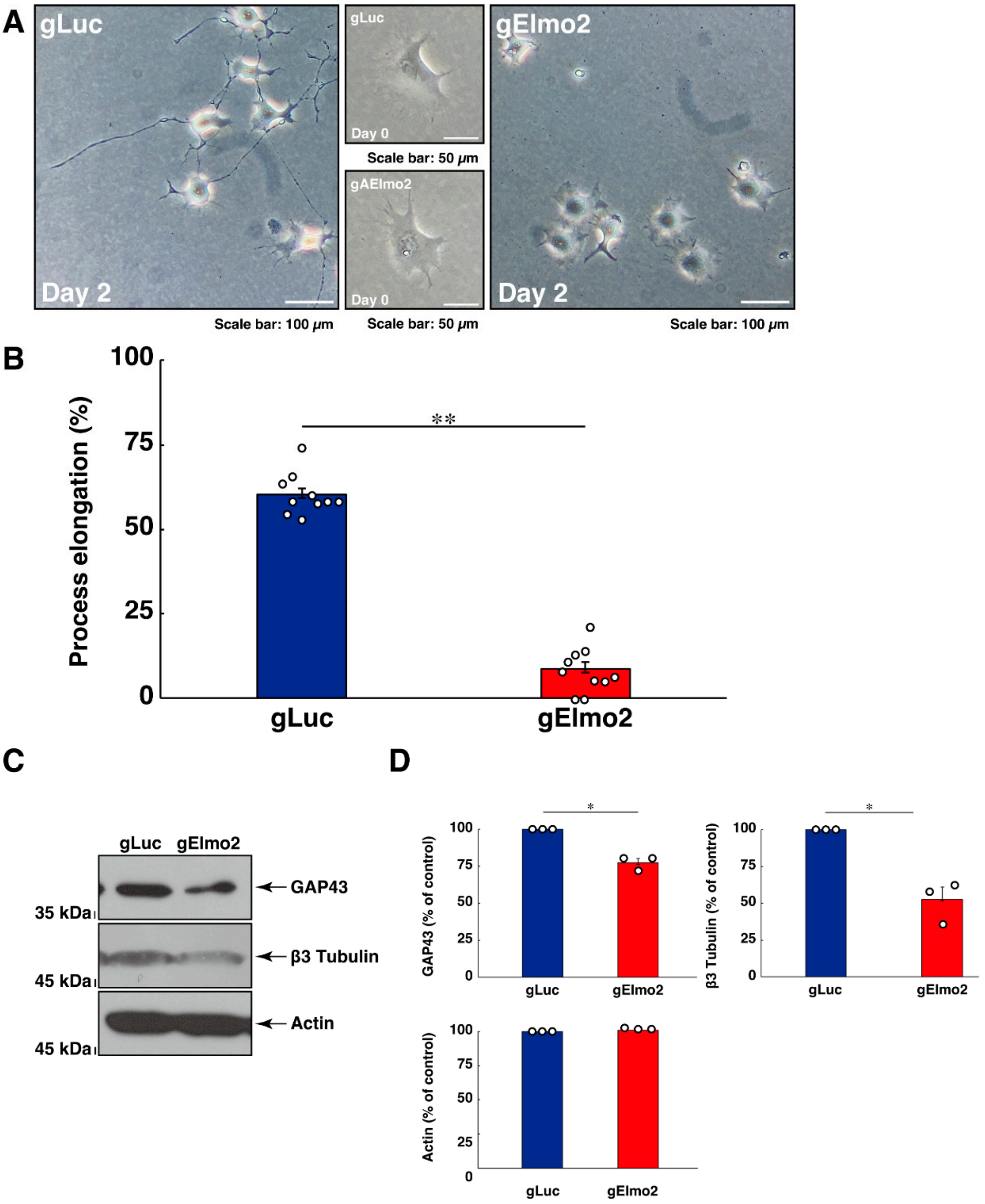

Figure S4. Effects of Elmo2 knockdown on process elongation in N1E-115 cells. (A, B) (A, B) Cells were transfected with the plasmid encoding CasRx and gRNA specific for Elmo2 or mock plasmid and allowed to differentiate for 0 or 2 days. Cells with processes exceeding a body length of two cells were counted as cells with neurite-like process elongation and statistically depicted in the graph (** $p < 0.01$; n = 10 fields). (C, D) The

lysates of cells at day 2 were immunoblotted with an antibody against a neuronal differentiation marker protein (GAP43 or beta3 tubulin) or an internal control protein actin. Their immunoreactive band intensities are statistically depicted (** $p < 0.01$; n = 3 blots).

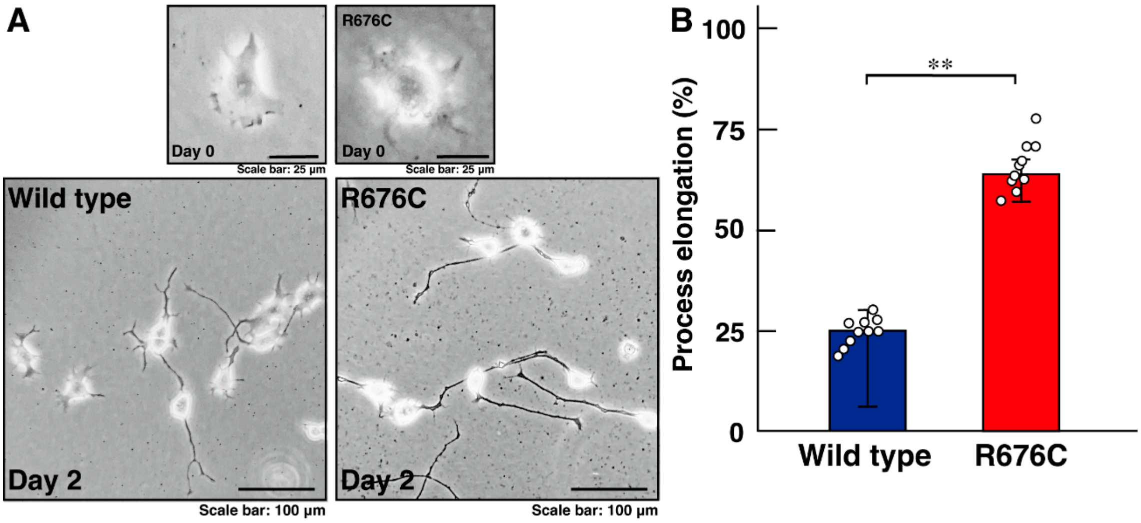

Figure S5. Effects of mutated Sema5A in process elongation in N1E-115 cells. (A, B) Cells harboring Sema5A with wild type (WT) or the R676C mutation were allowed to differentiate for 0 or 2 days. Cells with processes exceeding a body length of two cells are counted as cells with neurite-like process elongation and statistically shown in the graph (** $p < 0.01$; n = 10 fields).

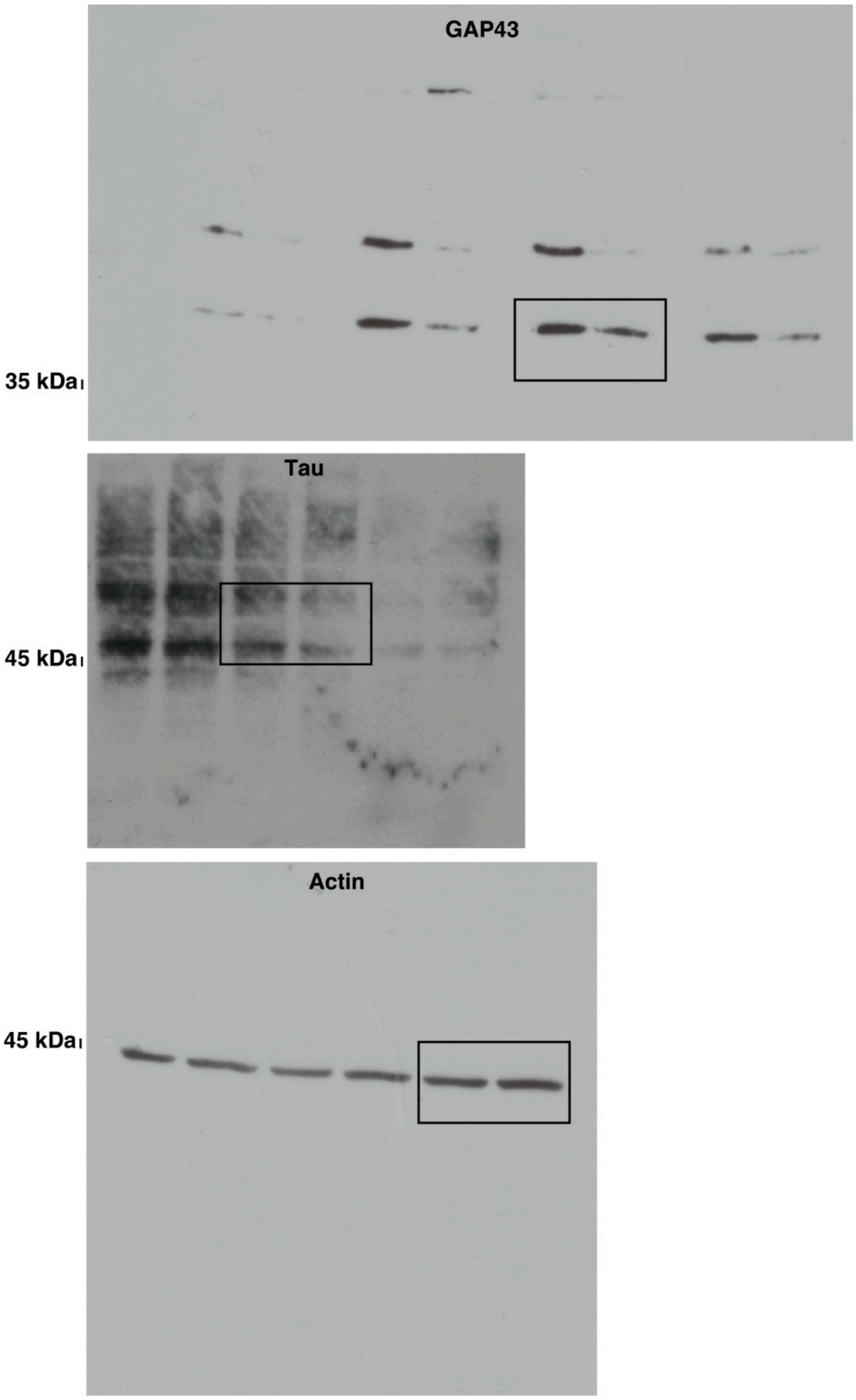

Figure S6. Full-size gel images of Figure 1 as computer-saved TIFF files.

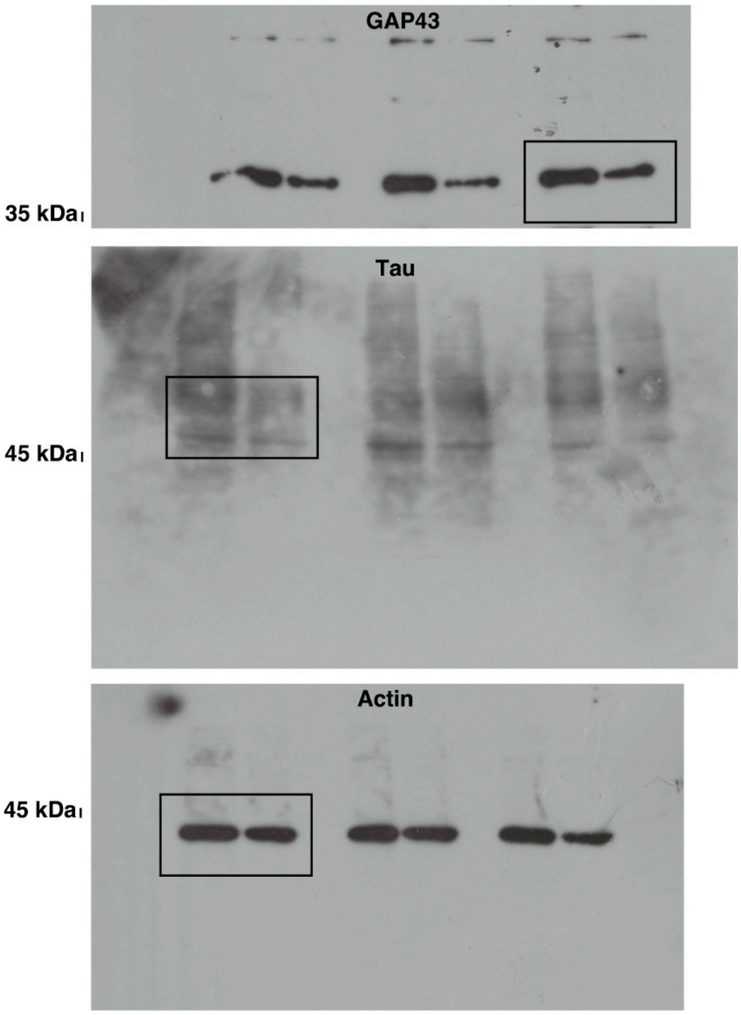

Figure S7. Full-size gel images of Figure 2 as computer-saved TIFF files.

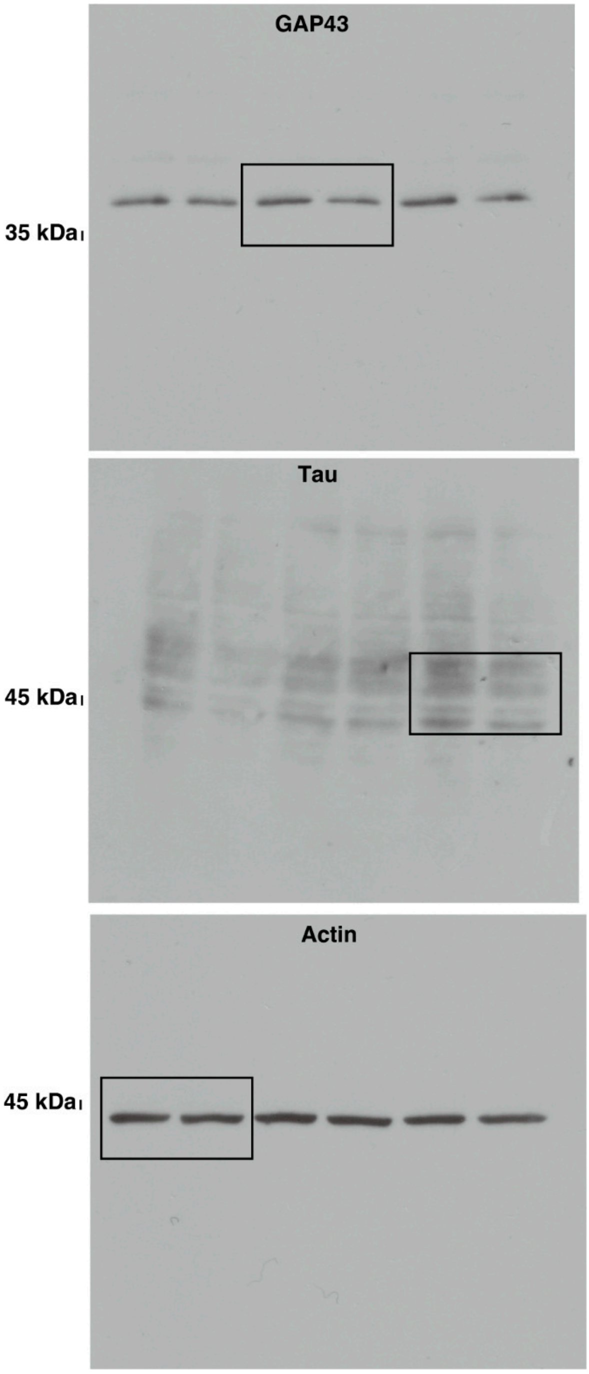

Figure S8. Full-size gel images of Figure 3 as computer-saved TIFF files.

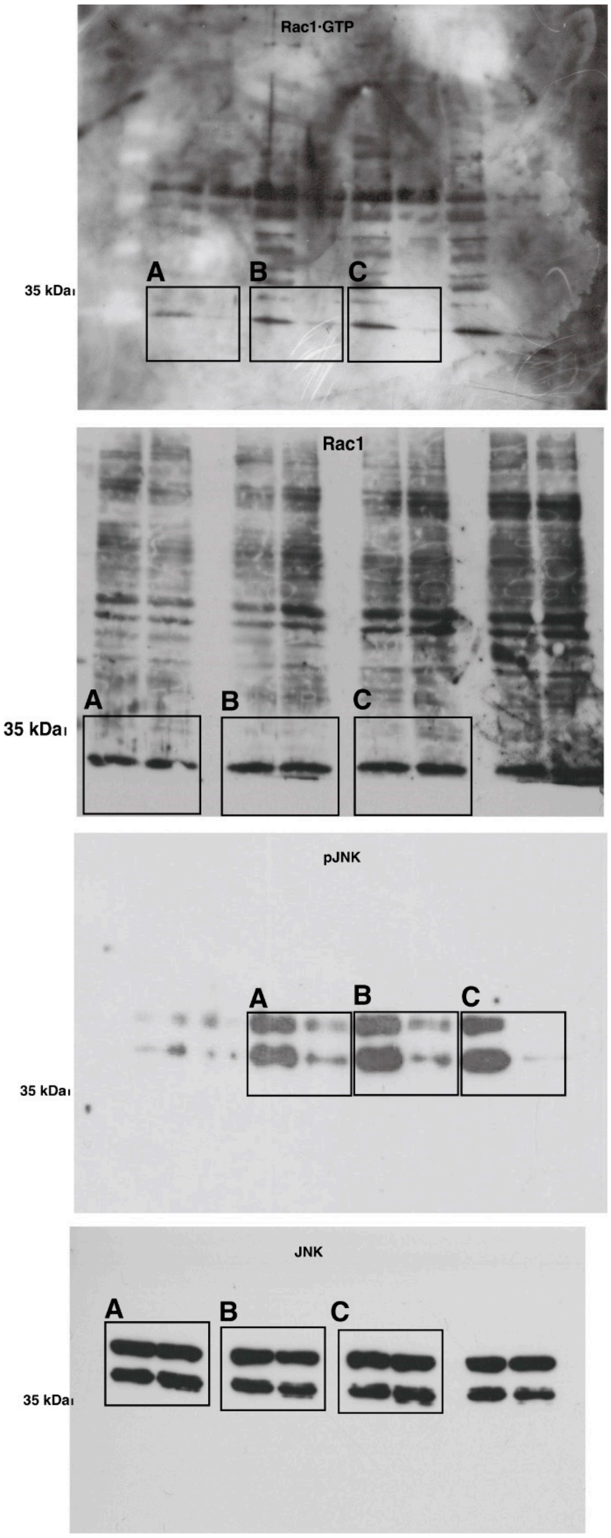

Figure S9. Full-size gel images of Figure 4 as computer-saved TIFF files.

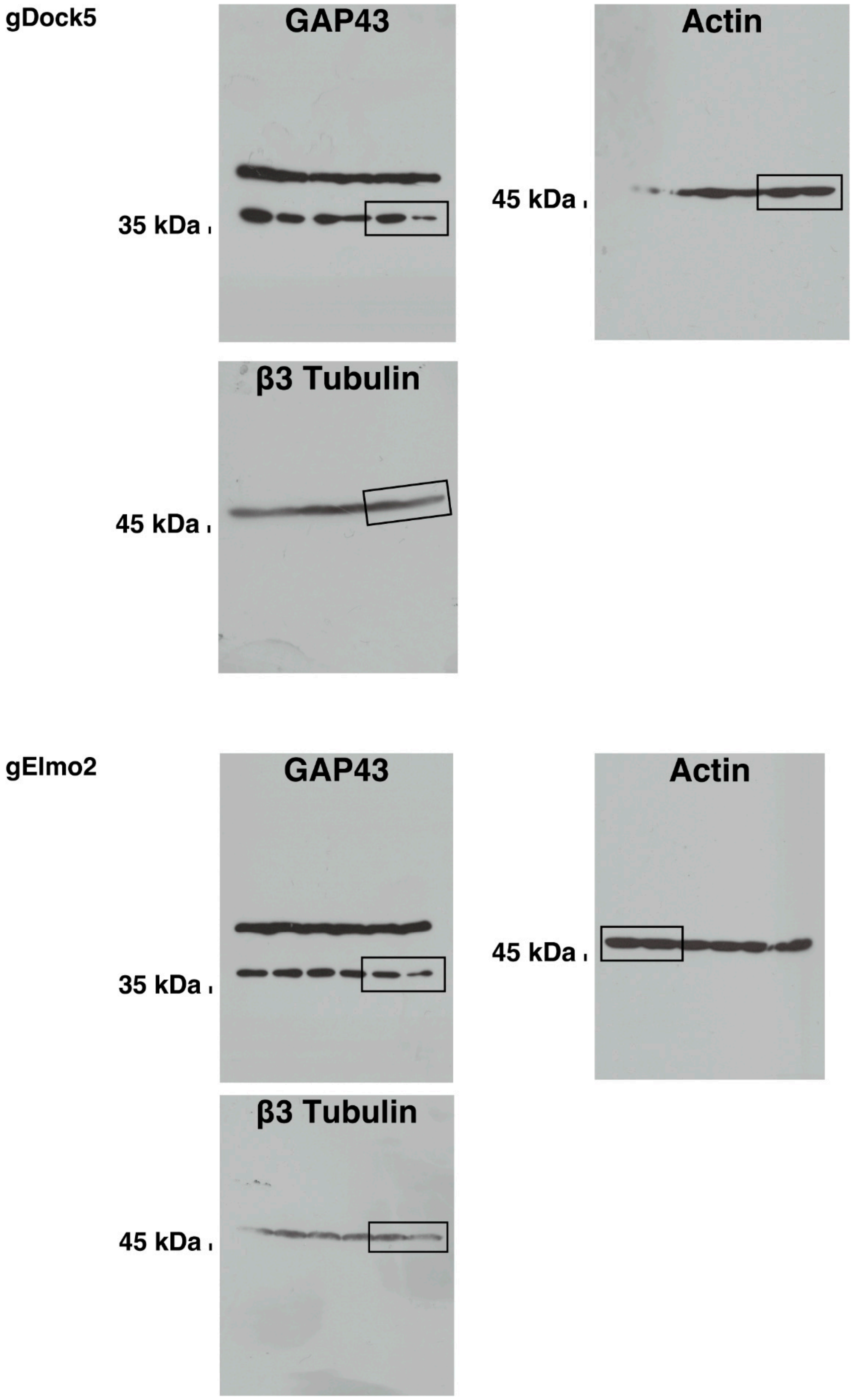

Figure S10. Full-size gel images of supplemental figures as computer-saved TIFF files.
